# The Challenges and Recommendations for Gestational Diabetes Mellitus Care in India: A Review

**DOI:** 10.3389/fendo.2017.00056

**Published:** 2017-03-24

**Authors:** Suman Morampudi, Gayathri Balasubramanian, Arun Gowda, Behsad Zomorodi, Anand Shanthanagowd Patil

**Affiliations:** ^1^FSRC (a Part of phamax), Bangalore, India

**Keywords:** gestational diabetes mellitus, India, health care, challenges, recommendations, gestational diabetes mellitus management

## Abstract

Gestational diabetes mellitus (GDM) is a primary concern in India affecting approximately five million women each year. Existing literature indicate that prediabetes and diabetes affect approximately six million births in India alone, of which 90% are due to GDM. Studies reveal that there is no consensus among physicians and health-care providers in India regarding management of GDM prepartum and postpartum despite available guidelines. Also, there is no consensus among physicians as to when a woman should undergo oral glucose tolerance test after delivery. This clearly shows that management of GDM is challenging and controversial in India due to conflicting guidelines and treatment protocols, despite availability of straightforward protocols for screening and management. Also, a collaborative approach remains a key for GDM management, as patient compliance and proper educational interventions promote better pregnancy outcomes. Management of GDM plays a pivotal role, as women with GDM have an increased chance of developing diabetes mellitus 5–10 years after pregnancy. Also, children born in GDM pregnancies face an increased risk for obesity and type 2 diabetes. The cornerstone for the management of GDM is glycemic control and quality nutritional intake. GDM management is complex in India, and existing challenges are multifactorial. However, there are little published data outlining these challenges. This review gives an account of some of the key challenges from self-management and health-care provider perspective. The recommendations in this review provide insights for building a more structured model for GDM care in India. This research has several practical applications. First, it points out to reaching a consensus on approaches for screening, diagnosis, and treatment of care across clinical practices in the nation that can aid in overcoming certain challenges observed. Second, it highlights the importance to build capacities and capabilities, especially in resource-limited settings. Health education among pregnant women remains a priority to resolve issues related to self-management. More broadly, further research, specifically qualitative is vital to determine forthcoming challenges with respect to patients, caregivers, providers, and policy makers and to provide solutions fitted to practice setting and demographic background.

## Background

Gestational diabetes mellitus (GDM) affects a significant proportion of pregnant women worldwide. GDM occurs when a woman’s pancreatic function is not sufficient to overcome the diabetogenic environment of pregnancy and causes high blood glucose levels due to the body’s extra demand for insulin (1). A variety of factors like age, diet, obesity, ethnicity, family history, history of GDM in previous pregnancy, macrosomia, essential hypertension or pregnancy-related hypertension, history of spontaneous abortions, and unexplained stillbirths cause an increased risk of glucose intolerance in pregnant women (2, 3). Globally, the prevalence of GDM varies widely depending on the population studied and the diagnostic test employed by researchers. Research suggests that GDM occurs in 2–10% of all pregnancies depending on the populations studied (4). In 2013, hyperglycemia in pregnancy was evident in approximately 17% of all live births across the world (5). Women with GDM have a 40–60% chance of developing diabetes mellitus over 5–10 years after pregnancy (6). Also, children born in GDM pregnancies face an increased risk for obesity and type 2 diabetes (7). Although GDM-associated mortality is rare, maternal and fetal mortality can occur when glucose levels are poorly controlled (8).

In India alone, GDM affects five million women each year (9). The Women in India with Gestational Diabetes Mellitus Strategy (WINGS) program, jointly conducted by the International Diabetes Federation (IDF), the Madras Diabetes Research Foundation, and the Abbott Fund, highlighted that prediabetes and diabetes affect approximately six million births in India alone, of which 90% are due to GDM (10). Hence, as per the WINGS study, only half of these women were tested postpartum for GDM, and there was no consensus among physicians as to when a woman should undergo oral glucose tolerance test after delivery despite available guidelines (10). This shows that the management of GDM is still challenging and controversial with conflicting guidelines and treatment protocols, despite availability of straightforward protocols for screening and management in the general population (10, 11). Also, effective communication among physicians, patients, and primary care providers is essential for GDM management, as patient compliance and proper educational interventions promote better pregnancy outcomes (12, 13). The cornerstone for the management of GDM is glycemic control and quality nutritional intake. However, GDM patients who fail to control their glucose levels through lifestyle modifications may require insulin. The national list of essential medicines in India includes insulin, which is considered as the gold standard for glycemic control during pregnancy (14). Therefore, it is affordable and easily accessible at the primary, secondary, and tertiary levels of health care in India. Nonetheless, there is no consensus on when to initiate insulin therapy for GDM in India (15). Research suggests that interventions such as self-management are effective in improving glycemic control, lowering health-care costs, and improving the quality of life in patients with diabetes (16, 17). However, the specific challenges in the effective self-management of GDM are not fully established, particularly among women with GDM in India, adding woes to already muddled provider management.

Existing studies show some of the challenges, knowledge gaps, and approach to GDM care. But, inefficiencies in establishing these challenges contribute to suboptimal patient outcomes (10, 13). Therefore, to confront the impediments in GDM management, it is also essential to understand the solutions that exist. The goal of this study was to review the available literature to establish the challenges and potential opportunities for improving GDM care in India and provide recommendations for the same.

## Methodology

The literature review focused on identifying the existing gaps and challenges in GDM management in India. The researchers identified studies that evaluated challenges in self-management and provider management of GDM in India. The researchers collected data through an extensive literature review process to present the consolidated information. First, key search terms like “Gestational diabetes,” “Gestational diabetes mellitus,” “GDM,” “epidemiology,” “challenges,” “barriers,” “management,” “screening,” “diagnosis,” “treatment,” “patient education,” “patient-centered care,” “recommendations,” “solutions,” and “India” were defined. Data were extracted using the search terms from diverse fields like health policies, obstetrics care, diabetes management, health-care access, health services research, and guidelines. The researchers then conducted iterative searches in electronic databases like Medline, PubMed, Cochrane Library, and references of selected articles to find relevant material on GDM in India. Since the subject demanded a thorough and systematic search, the data sources were not only limited to articles published in journals but also included gray literature. The sources for gray literature were as follows:

- Physician forums
- Institutional repositories like archives in hospital websites
- Physician association websites
- Popular Internet search engines
- Others (blogs, newsletters, and forums)

The search strategy was broad and sensitive to include as many relevant articles through subsequent manual screening. Again, the reference lists of relevant reviews and articles were thoroughly reviewed to ensure all important studies were covered. A thematic analysis approach was used to analyze the data retrieved in this review. The key challenges were segregated into categories that had similar ideas, concepts, or themes. The findings were then presented and discussed in detail.

## Results

**Figure 1:**
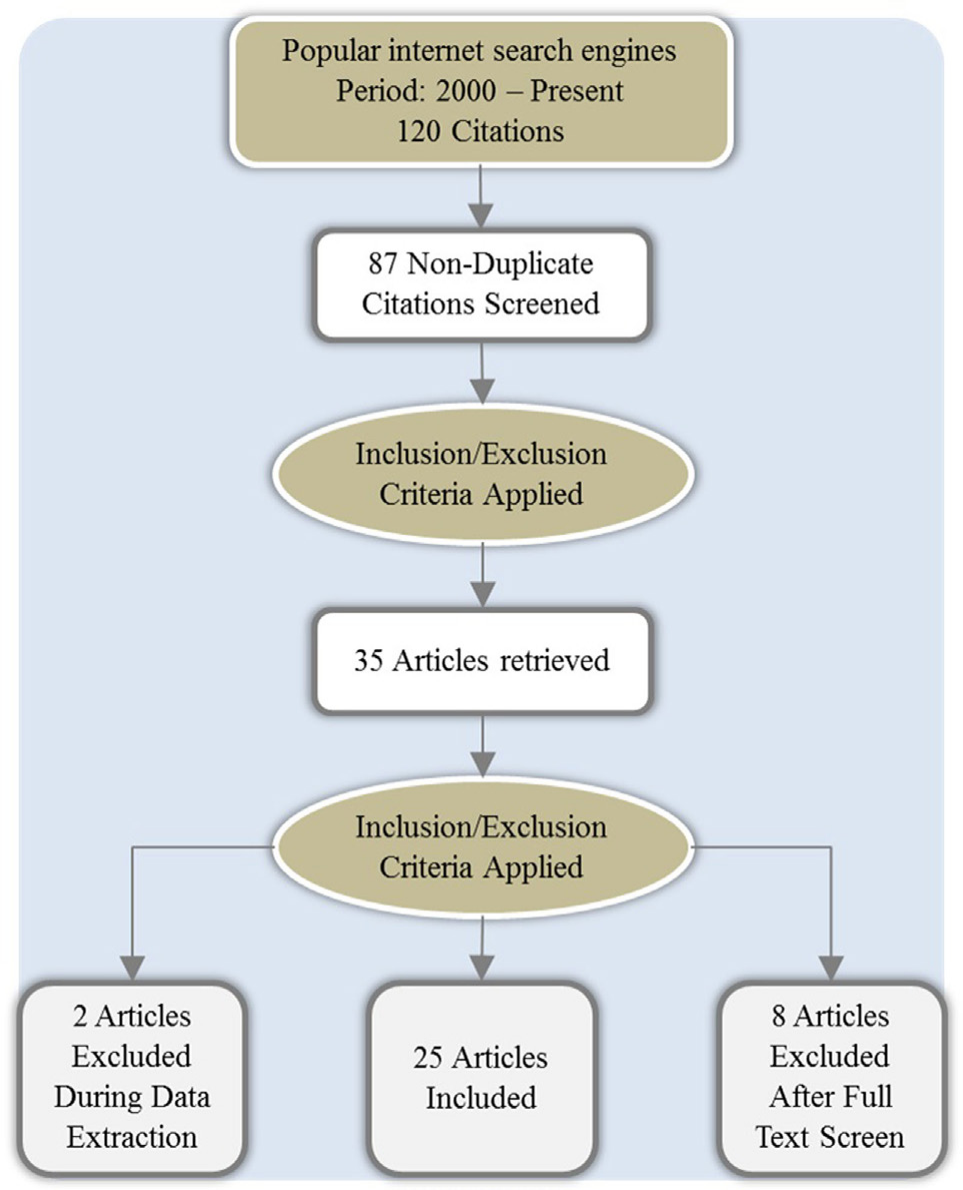
**The process of article selection for review**.

The researchers retrieved 87 articles from the initial search. Subsequently, the process of abstract sifting yielded 35 relevant articles that described GDM, its management, challenges, and other related information. The common reason for exclusion was non-relevance. Of these shortlisted articles, 25 citations met the inclusion criteria and were reviewed further (Figure 1).

### Characteristics of the Studies Included

We analyzed 25 studies for this review. Among the 25 studies on GDM, 6 (12, 13, 18–21) were on screening, 7 (10, 11, 13, 20, 22–24) on diagnosis, 9 (10, 11, 23, 25–30) on management, 8 (26–29, 31–34) on awareness, and 5 (31, 35–38) on epidemiology. The increase in the total number of studies is due to the single studies that focused on more than one topic. Of the 25, 8 studies (12, 20, 21, 25, 27, 28, 30, 32) were literature reviews/editorials, 13 (10, 13, 19, 22, 26, 31, 35–38) epidemiological studies and surveys, and 4 (11, 18, 23, 34) reports/practice guidelines. The characteristics of these studies are also listed in Table 1.

**Table 1:** **Characteristics of studies included**.

	Author(s)	Study objective(s)	Study design	Area of study
1	Mahalakshmi et al.	To obtain information on existing practices in the diagnosis and management of gestational diabetes mellitus (GDM) among physicians/diabetologists/endocrinologists and obstetricians/gynecologists in India	Epidemiological study (online/in–person surveys) (10)	Current practices in diagnosis and management
2	Seshiah et al.	To outline Indian guidelines for diagnosis and management of GDM	Practice guidelines (11)	Diagnosis and management
3	Mohan et al.	To assess the criteria to be used to diagnose GDM	Review (12)	Screening
4	Balaji et al.	To elucidate a test that is casual and reliable to diagnose GDM	Epidemiological study (prospective cohort study) (13)	Screening and diagnosis
5	Seshiah et al.	To outline the necessity of screening for all Indian pregnant women	Practice guidelines (18)	Screening
6	Raja et al.	To estimate the prevalence of GDM and various sociodemographic factors of the studied subjects	Epidemiological study (community-based cross sectional study) (35)	Epidemiology
7	Seshiah et al.	Presents updated clinical evidence with expert inputs in the context of Indian clinical practice	Editorial (25)	Guidelines management
8	Jain et al.	To study the effect of glucose levels on maternal and fetal outcomes	Epidemiological study (prospective cohort study) (36)	Epidemiology
9	Seshiah et al.	No data are available about the prevalence of glucose intolerance during pregnancy in our country, and hence a study was undertaken on this aspect	Epidemiological study (prospective study) (37)	Epidemiology
10	Arora et al.	To determine the prevalence and risk factors of GDM comparing the previous World Health Organization (WHO) 1999 criteria to the WHO 2013 criteria in North India	Epidemiological study (cross-sectional design with a questionnaire) (38)	Epidemiology
11	Kayal et al.	Women in India with Gestational Diabetes Mellitus Strategy (WINGS): methodology and development of model of care for GDM (WINGS 4)	Epidemiological study (situational analysis) (26)	Management and awareness
12	Jindal et al.	To study the prevalence of glucose intolerance at 6 weeks postpartum in Indian women with GDM diagnosed according to ADA criteria	Epidemiological study (longitudinal study) (31)	Epidemiology and awareness
13	Mohan et al.	The aim of this study was to compare the Diabetes in Pregnancy Study Group of India (DIPSI) criteria with the WHO 1999 and the International Association of the Diabetes and Pregnancy Study Groups (IADPSG) criteria for GDM	Epidemiological study (prospective study) (19)	Screening
14	Pulkit et al	Our objective was to study the implications of implementing the IADPSG guidelines or DIPSI guidelines for screening and diagnosis of GDM in Indian population. Another objective was to evaluate the importance of isolated fasting glucose, which is the main difference between the two guidelines	Epidemiological study (retro-prospective study) (22)	Diagnostic criteria
15	Madhab et al.	To advocate policy change for GDM in India	Review (32)	Awareness
16	Mithal et al	To understand the impact of GDM	Editorial (27)	Awareness and management
17	Poomalar	To understand changing trends in management of GDM	Review (28)	Awareness and management
18	International Diabetes Federation	This project aimed to develop a context-adapted model approach to care in low-resource settings, which confronts the widespread challenges in GDM screening and management	Epidemiological study (situational analysis) (29)	Awareness and management
19	National Health Mission, Government of India	Guidance note on National Guidelines for Diagnosis & Management of GDM	Practice guidelines (23)	Diagnosis and management
20	Bhavadharini et al.	This review intends to provide an overview of the evolution of the screening and diagnostic criteria for GDM	Review (20)	Screening and diagnostic criteria
21	Sharma et al.	To elucidate an evidence-based single glucose challenge test to diagnose GDM	Epidemiological study (cohort study) (24)	Diagnosis
22	Gupta and Kalra	To evaluate methods that improve postpartum screening rates	Review (21)	Screening
23	Kalra et al.	To elucidate psychological effects of GDM on pregnant women	Editorial (30)	Management
24	Shriraam et al.	Awareness of GDM among antenatal women in a primary health center in South India	Epidemiological study (survey) (33)	Awareness
25	Jagran Prakashan Limited	Annual report (2010–2011) with all the initiatives that Jagran Pehel has embarked upon in the particular year	Report (34)	Awareness

Overall, three studies furnished data on self-monitoring of glucose levels and regulation of diet and exercise, adherence to medications, and periodic follow-up with the health-care provider. Similarly, 12 studies discussed GDM awareness among patients, management guidelines (prepartum to postpartum), patient compliance, and the need for educational and behavioral counseling. The data from 13 studies highlighted GDM care in India and discussed cultural tailoring of interventions, inconsistencies in screening, diagnosis and management guidelines across practices, individualized assessment and reassessment, and use of treatment algorithms by various health-care providers.

### Analysis

**Figure 2:**
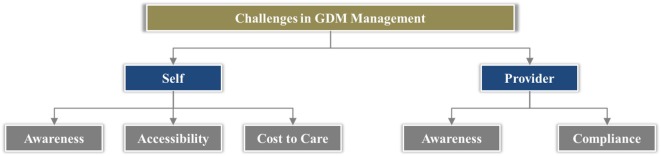
**Challenges in GDM management**.

We analyzed data from these studies and categorized the challenges under two key themes covering the vital aspects of GDM care (Figure 2):

- Challenges in self-management
- Challenges in provider management

In addition, we provided recommendations for the outlined challenges based on relevant information extracted from these studies (Table 2).

**Table 2:** **GDM management: challenges and recommendations**.

Themes	Sub-themes	Challenges and recommendations
Challenges in self-management	Awareness	**Disease**: one key challenge in India was inadequate awareness about the disease among the public. Hence, creating gestational diabetes mellitus (GDM) awareness among public is an imperative
		**Recommendations**: increased knowledge about GDM among antenatal women pave way to a healthy lifestyle, better health-care-seeking pattern and self-care and thus prevention and early diagnosis of the disease (33). Television/radio, neighbors/friends, and family members seemed to be preferred sources of GDM awareness compared to doctors, health-care workers, or hospital charts/boards from which lesser proportion of women sought information from Ref. (33). However, this was the rural scenario. Many researchers opined that mass media should be properly utilized to increase service utilization for GDM by showcasing evidence, raising awareness, and creating public opinion through dialog and discussion. Several programs were implemented to increase awareness about GDM among pregnant women in India (27). One such campaign was Jagran Pehel (a social initiative by Jagran Prakashan Limited), which was supported by the World Diabetes Foundation. The program reached over half a million people in seven districts of four states in northern India. Information on GDM was exchanged with over 3.7 million people using the mass media. Health-care workers have to play a greater role in creating awareness on GDM among antenatal women (34)
		**Management**: many women are unaware of the importance of GDM management. Upon diagnosis with GDM, doctors advise either lifestyle or medical interventions. Self-management is also vital. GDM patients are closely monitored until delivery and even beyond. However, such interventions are not recognized by many patients, especially in the rural sector. A study conducted among primary health centers showed that women were well aware of the importance of diet and exercise in GDM and the probability of untreated GDM posing a risk to the unborn child. However, knowledge about the risk factors and course of GDM and that the women diagnosed with GDM are at an increased risk for future type 2 diabetes was low (33). Insufficient information on maintaining blood glucose within the recommended range is a risk factor in GDM. Also, lack of awareness about GDM and its complications, late contact with the health-care provider, costs involved in treatment, and the myths surrounding insulin use are some serious threats to GDM care (26). Many women with GDM experience pregnancy-related complications including high blood pressure, large birth weight babies, and obstructed labor (29). Globally, data show that children of mothers with uncontrolled diabetes—either pre-existing or originating during pregnancy—are four to eight times more likely to develop diabetes in later life compared to their siblings born in a non-GDM pregnancy (27, 31)
		**Recommendations**: knowledge about the risk factors and the consequences of untreated GDM are highly important for women to take proper precautions and self-care (33). As GDM is associated with adverse fetal, neonatal, and long-term complications, there is a need to improve awareness among women for preventing and managing GDM (28). Lifestyle interventions or metformin are offered to women with GDM. Patients must be aware of the importance of self-management and be educated about the same. Information guides and charts on food, exercise, and usage of glucose monitors aid in self-management (26, 28)
		**Social taboos and myths**: there are social taboos and myths in Indian communities that interfere with the effective management of GDM. Particularly, in rural areas, a diagnosis of GDM can enhance stress in pregnant women due to misconceptions among family members leading to conflicts. Further, given the high unawareness about GDM, the in-laws of the woman may accuse her off keeping the ailment a secret from before marriage. Other accusations can be that the GDM is the patient’s fault, because of eating too much or not performing domestic chores (30). Many false perceptions interrupt the doctors’ recommended lifestyle interventions such as a pregnant women should give into her cravings for sweets if she will listen to the doctor and exercise can harm the baby. This can have a deleterious effect on the patient’s health and the fetus. Also, a lack of awareness about the myths surrounding insulin use also poses a serious threat to GDM care (26)
		**Recommendations**: many studies suggest the need to educate pregnant women on issues related to GDM like the importance of regular monitoring, follow-up, information from the right sources, reliance on health-care providers, and more (26, 33). Lifestyle modifications like increase in physical activity, decrease in consumption of sweetened beverages, and high-energy dense food item should be introduced earlier and continued throughout the life as advised by health-care providers

	Accessibility	**Management**: health-care resources are insufficient. This results in a large population being hesitant to access health care for diseases with not so “obvious” implications like GDM (12). There are several challenges in the screening and diagnosis for GDM in India. Some of the major patient-related barriers are late contact with the health-care system as pregnant women have to travel long distances to meet the doctor, women not routinely attending antenatal check-up in a fasting state, and lack of awareness about GDM and its complications. Hence, making women undergo the test in the fasting state could be a challenge (12, 20)
		**Recommendations**: direct home visits, screening with hemoglobin A1C levels and self-administered oral glucose tolerance test may lead to increased testing rates and may be a part of the solution of this complex problem (21). The need to train manpower and mobilize resources to improve access also is recommended (27)

	Cost to care	**Cost to care**: costs of care may be a concern for patients, especially from the rural sectors
		**Recommendations**: studies suggest the need for a cost-effective, evidence-based and patient-friendly approach to the diagnosis and management of GDM (24). The best strategies for screening and diagnosis should be based on the cost and availability of the local health facilities (20). Many women with GDM also develop type 2 diabetes, resulting in further health-care complications and costs (29). The DIPSI criterion is a one-step cost-effective and evidence-based procedure to diagnose GDM in any socioeconomic setting (12, 13, 23). The Indian Ministry of Health introduced free screening for GDM among the five services offered to pregnant women below the poverty line in the National Rural Health Mission program. Several state governments like Bihar, Delhi, Jharkhand, and Punjab have pledged similar initiatives for GDM; the Government of Tamil Nadu is already implementing such a policy (32)

Challenges in provider management	Awareness	**Guidelines and criteria**: screening, diagnosis, and treatment: the lack of trained health-care professionals and phlebotomists, scarcity of diagnostic facilities and standardized laboratories, storage and transport of blood samples, etc. are a few barriers to screening and diagnosis in low-resource settings (20). Also, there is inconsistency in the guidelines followed by doctors across the nation. Women with a history of GDM are greatly at a risk of subsequent diabetes and should be screened for prediabetes or diabetes. Health-care providers need to be aware of this
		**Recommendations**: the need to train manpower and mobilize resources to improve access is recommended (27). Universal screening for GDM should be followed, as women of Asian origin, especially Indians, are at a higher risk to develop GDM and subsequent type 2 diabetes (35). Blood sugar levels can indicate maternal and perinatal morbidity and mortality in GDM cases. Therefore, there is a need for unified diagnostic criteria and guidelines to create awareness among all Indian laboratories (19, 22, 23, 36, 38). DIPSI recommends a 75 g oral glucose load and a venous blood sample after 2 h to estimate plasma glucose for a pregnant woman visiting an antenatal clinic in the fasting state. Screening is recommended between 24 and 28 weeks of gestation. A team approach is ideal for managing women with GDM (18, 25). The maternal health and fetal outcome depends upon the care by the committed team of diabetologists, obstetricians, and neonatologists; also, health-care providers need to have a collaborative approach (18, 25). A short-term intensive care gives a long-term pay off in the primary prevention of obesity, IGT, and diabetes in the offspring, as the preventive medicine starts before birth (18)

	Compliance	**Patient compliance**: a major challenge for doctors is that patients do not comply with the treatment or the recommended number of follow-up visits due to various reasons
		**Recommendations**: general awareness about GDM and risk factors, diagnosis, treatment, and consequences of GDM may improve treatment compliance and self-management in patients (33). The increasing prevalence of GDM and its comorbidities among pregnant women demands the need to educate patients on compliance (34)

## Discussion

Before expounding the challenges and recommendations to GDM care, it is necessary to acquaint with the health-care delivery system in India.

### Overview of Health System in India

**Figure 3:**
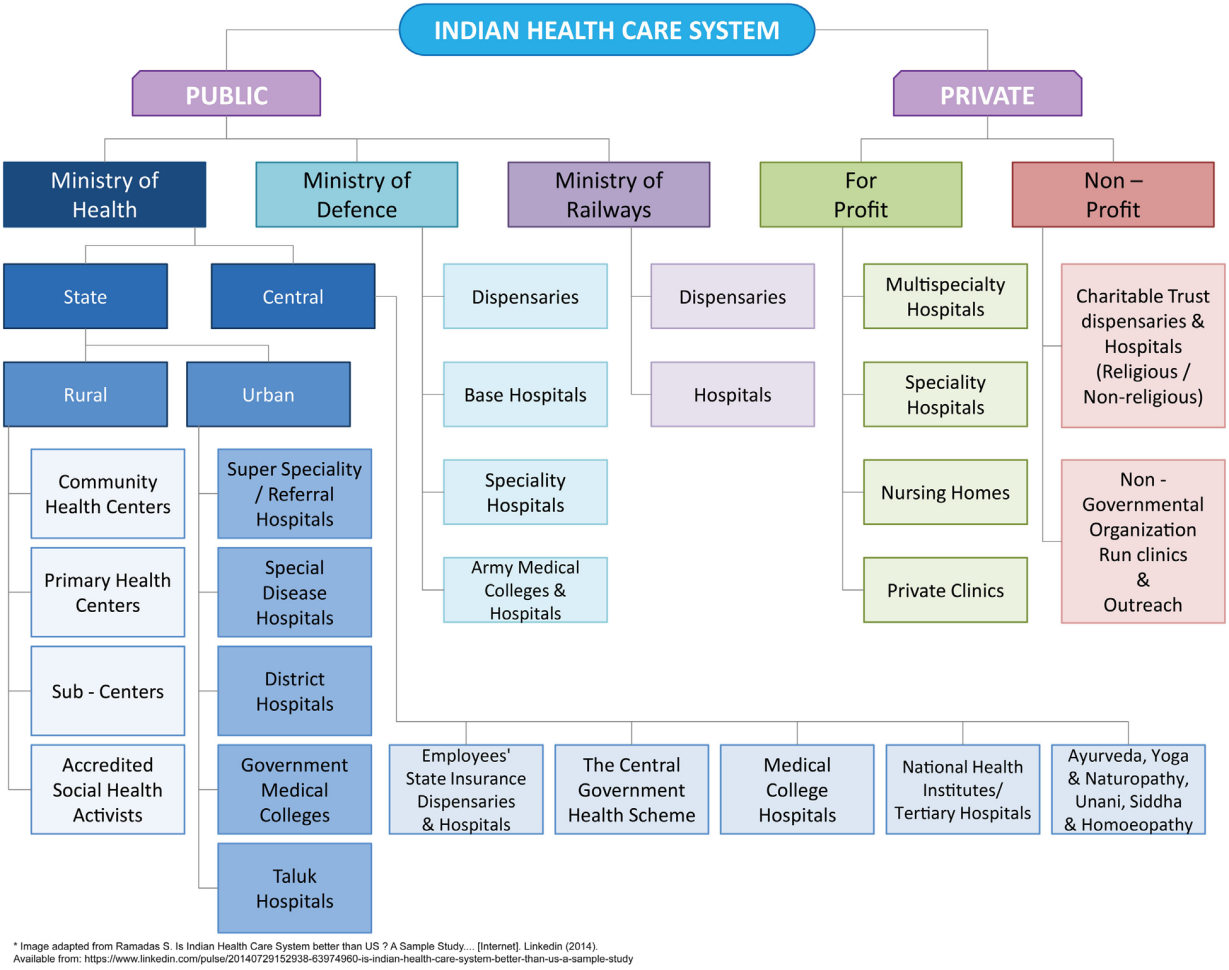
**An overview of Indian healthcare system**.

Health-care system in India is categorized into two major sectors: public and private (Figure 3). The public health-care system chiefly includes secondary and tertiary care institutions in key cities and primary health-care centers in rural areas (39). On the other hand, the private sector provides majority of care through secondary, tertiary, and quaternary institutions with a major focus in tier I and tier II cities (39). Thus, the health-care infrastructure in urban and rural India is not evenly distributed and not at proximity, mostly in case of rural areas. Although, health-care services in the public sector are offered free of charge to all citizens, people often end up with high out-of-pocket expenditures because they often prefer services from private health care due to quality of care (40). Further, the health-care costs vary within the private sector based on the facilities and care offered, leading to issues related to affordability. On the whole, the total expenditure for health in India was 4.7% of GDP in 2014 of which public and out-of-pocket health expenses were 30 and 62.4%, respectively (41).

Apart from public and private health-care providers, non-profit organizations and non-governmental organizations also play a pivotal role for GDM-related advocacy. Currently, organizations, both national and international, such as IDF, Bill and Melinda Gates Foundation, Jagran Pehel, Women and Children First, Public Health Foundation of India, and others work toward promoting maternal and child health (9, 42–45). Another coalition in India that strives toward the same goal is the reproductive, maternal, newborn, child, and adolescent health (RMNCH+A) coalition, which is led by Save the Children India under the support of the Government (46). The coalition includes central and state government agencies, academia, research and training institutes, health-care professional associations, local bodies (Panchayats and Nagarpalikas), non-governmental organizations, civil society organizations, faith-based organizations, media, corporate organizations, bilateral and multilateral donors, and United Nations agencies (46). RMNCH+A aims to work more effectively in collaboration with multiple stakeholders to enhance joint action and accountability and to support the implementation of national commitments and policies (46). In 2005, a global alliance “The Partnership for Maternal, Newborn & Child Health” of more than 700 organizations in 77 countries representing the sexual, reproductive, maternal, newborn, child, and adolescent health communities, as well as health influencing sectors, was established (47). This partnership provides a platform for organizations in India to align objectives, strategies, and resources and to achieve consensus on interventions to improve maternal, newborn, child, and adolescent health (47).

To establish the existing challenges and recommendations for GDM care in India, the study researchers performed a narrative review using available literature on GDM in India. The key challenges identified during this review with regard to self-management and provider management included awareness, accessibility, compliance issues, and cultural context of care. The study researchers discussed the identified challenges and suggested recommendations based on the findings from the literature. Also, the researchers have provided additional recommendations in context of the findings after reviewing the most recent guideline documents and several international publications to suggest the best practices for GDM management.

### Challenges in Self-Management

Some major challenges in the self-management of GDM were social taboos, cultural habits, GDM awareness, adherence, lack of patient motivation, screening costs, and transportation to nearest centers. Many of these challenges do not require adequate resources, but simple policy changes to improve GDM awareness. In India, the growing concern of family and friends for a pregnant woman and her baby is a major factor. Almost everyone within the family has an opinion. This can confuse and complicate the pregnant woman’s decisions to follow the health-care professional’s advice. The lack of information in communities and sometimes cultural perspectives are barriers to GDM care. Myths like “exercise harms the baby” and “pregnant mothers must consume food for two” cause negative strongholds in pregnant women, preventing them from following the instructions of their health-care professionals to exercise and adhere to a certain diet. Some patients are even reluctant to ask questions, suggesting poor interaction with the health-care providers leading to misconceptions (48, 49). Hence, many women remain sedentary during pregnancy because of these perceived barriers. Many women crave certain food items during pregnancy, and the temptation for food that is not necessarily nutritious, especially carbohydrates, is a major obstacle to adherence (48, 49). Although studies suggest that exercise is good in pregnant women, in reality, compliance to diet, exercise, and medications is a major challenge to care in GDM patients, given one’s cultural habits. There is a need to increase awareness among patients on the importance of diet, exercise, and medication while educating them on myths and health facts (48, 49).

The lack of awareness on GDM among patients is a major hurdle to its successful management. Majority of patients know little about blood glucose monitoring and adherence to treatment. This affects the treatment process and benefits. The limited knowledge on dietary issues and disease management causes malnutrition for both the mother and the fetus due to poor glucose control (49). Therefore, it is vital to educate patients about the disease, its complications, management strategies, and the importance of adherence. Previous research also suggests that information is crucial for patient adherence to treatment and self-management of the disease (48). However, the sources of information must be reliable. At all times, health-care providers are the greatest source of valid, reliable, and comprehensive information on GDM and its management.

Although pregnant women try to meet their nutritional needs and are careful about their own health, they disregard the importance of medicine. Studies show that GDM patients often encountered fear and emotional disturbances when informed about the consequences of the disease (48, 49). So, educating them on the importance of medicine and adhering to dietary recommendations improves their medicine intake and makes them more likely to embrace a healthier routine (49, 50).

Adding to the disease burden, the costs associated with GDM management are a barrier for pregnant women seeking consultation with the doctor and antenatal care. In India, many patients do not have health insurance and everything is out of pocket for major health services during pregnancy. Equipment such as glucometers and related supplies, medications, and diet modifications cause financial burdens. Low income, limited availability of public health centers at close proximity, and periodic travel to hospital for follow-ups also add to the financial burden of pregnant women in rural areas. Thus, there is a need to increase access to health-care services to reduce GDM burden in India.

### Challenges in Provider Management

Some of the major challenges encountered in provider management were getting pregnant women to visit in a fasting state, getting blood samples, lack of trained phlebotomists, and standardized laboratories for blood glucose estimations, conflicting guidelines across practices and patient compliance (12). Screening should be made mandatory for all pregnant women due to the high prevalence of GDM among Indian women. The GDM status should be a part of a physician’s routine history assessment, irrespective of the pregnancy or parity, as GDM is a precursor for type 2 diabetes (51). Therefore, early screening and diagnosis can prevent obesity, impaired glucose tolerance, and diabetes in the progenies and mothers (52).

Most researchers shared the same opinion that GDM screening is widely deliberated, more specifically on selective versus universal screening, timing of testing, methods, and the diagnostic criteria. Some of the controversies surrounding this subject remain unresolved (53). First, getting a pregnant woman to undergo a GDM screening in a fasting state is challenging, particularly in a country like India. Second, multiple screening tests to diagnose GDM coupled with factors such as low awareness, less accessibility, and low affordability are a concern in resource-limited settings. Therefore, the World Health Organization 1999 criteria, which require only a single sample in comparison to the three samples required by the International Association of the Diabetes and Pregnancy Study Groups (IADPSG) criteria and four samples required by the Carpenter and Coustan criteria, became very popular in India initially (54). In 2014, the Ministry of Health and Family Welfare had developed technical and operational guidelines for identification and management of GDM in India. The national guidelines for diagnosis and management of GDM recommend a single-step procedure using 75 g oral glucose in a fasting or a non-fasting state and measuring plasma glucose 2 h postingestion (23). This one-step procedure to diagnose GDM is preferred as it is simple, economical, and feasible. Although the criteria for screening and diagnosis are established, uncertainty still exists on the execution methods.

Screening remains vital to prevent GDM-related complications during perinatal period and delivery. Evidence suggests that universal screening improves pregnancy outcomes compared to selective screening (55). Many guidelines recommend universal screening, while others exempt patients categorized as low-risk groups. Low-risk patients are younger than 25 years, have normal body weight, have no first-degree relatives with diabetes, show no history of abnormal glucose metabolism or poor obstetric outcomes, and are not from an ethnic group with a high diabetes prevalence (Hispanic American, Native American, Asian American, African American, and Pacific Islander) (56). Contrarily, a few researchers argue that selective screening based on the clinical characteristics of a pregnant woman facilitates efficient screening for GDM (57). The risk for GDM varies among different pregnant women based on marked obesity, previous history of GDM, glycosuria, or family history of diabetes. Nonetheless, all pregnant women should be screened for GDM during their first antenatal visit (51). Although few experts object to the screening of low-risk patients routinely, research suggests that non-screening omits approximately 4% patients with GDM (58). Universal screening for GDM detects more cases and improves maternal and neonatal prognosis compared to selective screening (59). The US Preventive Services Task Force (2014) also recommends that all asymptomatic pregnant women be screened for GDM after 24 weeks of gestation (53).

Many International medical associations like the American College of Obstetricians and Gynecologists, the American Endocrinology Society, the Canadian Diabetes Association, Australian Diabetes Association, and the Diabetes in Pregnancy Study Group of India also endorse that screening for GDM should be universal. Although certain organizations like the American Diabetes Association (ADA) and the National Institute for Health and Care Excellence recommend selective screening, even they agree that Asians, especially Indians, are a high-risk ethnic group (12). Therefore, Indian pregnant women should be universally screened at their first registration. The benefits of universal screening cannot be ignored in the long term given the high prevalence of GDM across India in spite of the increased screening costs for the government and individuals (51, 59).

Gestational diabetes mellitus patients ideally need counseling about the disease from the time of diagnosis. The education should cover recommended diet, exercise, treatment, self-care, and monitoring. There is also a need to train them efficiently to use monitoring equipment for self-management. The patients’ family members should also be educated on emotional and psychological support, the needs associated with GDM, and general pregnancy care. It is vital to highlight the importance of antenatal care and that GDM management requires a holistic support system. The first line of management for women with GDM is medical nutrition therapy (MNT) or dietary modification, followed by physical activity and monitoring of blood glucose levels. MNT reduces pregnancy and perinatal complications and brings glycemic control (8). A study by Jovanovic-Peterson et al. showed that exercise (arm ergometer training) coupled with dietary modifications improved glycemic control compared to dietary modifications alone (60). Home-based exercise training improved capillary glucose profile in women with gestational diabetes. A study by Halse et al. showed that home-based cycling is effective in maintaining daily postprandial normal glycemic levels in women with diet-controlled GDM. Further, the researchers noticed that the compliance to the supervised exercise training was good, and capillary glucose concentration reduced in response to each cycling session despite high consumption of dietary carbohydrates (61). Exercise proved to be beneficial in GDM patients, but there are no guidelines for the same until recently. In 2015, Padayachee and Coombes drafted the first guidelines on exercise for GDM management (62).

Further, in women prescribed with insulin, hospitalization proved to be effective in achieving treatment compliance to regulate glucose levels (48). Although it is not possible to hospitalize every GDM women to regulate glucose levels in resource-limited settings, it can be inferred that managing GDM requires a collaborative approach. Well-trained professionals play a crucial role in screening, diagnosis, and treatment. A team approach is ideal to manage women with GDM usually comprising an obstetrician, diabetologist/endocrinologist, health education provider, dietitian, and neonatologists/pediatrician (18).

Intensive monitoring, diet, and insulin are the cornerstones of GDM management. Until there is absolute evidence that ignoring maternal hyperglycemia is acceptable when the fetal growth patterns appear normal on the ultrasonogram, it is prudent to achieve and maintain normal glycemic levels in every pregnancy complicated by GDM. The maternal health and fetal outcome depend on the care by a committed team of health-care professionals. A short-term intensive care gives long-term benefits in preventing obesity, impaired glucose tolerance and diabetes in the offspring, as the preventive medicine starts before birth. The future solutions offered by physicians should focus on tangible and simple ways to better monitor patients and standardize care across practice.

## Conclusion

In India, GDM management is complex, and existing challenges are multifactorial. This review established some of the key challenges from self-management and health-care provider perspective. The recommendations provided in the literature and by this study researchers pave way for building a more structured model for GDM care in India. Reaching a consensus on approaches for screening, diagnosis, and treatment of care across clinical practices in the nation can aid in overcoming certain challenges observed. It is important to build capacities and capabilities, especially in resource-limited settings. Health education among pregnant women remains a priority to resolve issues related to self-management. To conclude, periodic research, specifically qualitative, can elicit forthcoming challenges with respect to patients, caregivers, providers, and policy makers and provide solutions tailored to practice setting and demographic background.

## Author Contributions

SM, GB, AG, BZ, and AP declare that we have made substantial contributions to the conception or design of the work or data collection, analysis, or interpretation of data for the work; drafting the work or revising it critically for important intellectual content; final approval of the version to be published; and agree to be accountable for all aspects of the work in ensuring that questions related to the accuracy or integrity of any part of the work are appropriately investigated and resolved.

## Conflict of Interest Statement

The authors declare that the research was conducted in the absence of any commercial or financial relationships that could be construed as a potential conflict of interest. The reviewer RM and handling Editor declared their shared affiliation, and the handling Editor states that the process nevertheless met the standards of a fair and objective review.
